# Outcome after allogeneic stem cell transplantation with haploidentical versus HLA-matched donors in patients with higher-risk MDS

**DOI:** 10.1038/s41409-023-01931-7

**Published:** 2023-02-11

**Authors:** Claire Michel, Marie Robin, Stephane Morisset, Didier Blaise, Johan Maertens, Patrice Chevalier, Cristina Castilla-Llorente, Edouard Forcade, Patrice Ceballos, Ibrahim Yakoug-Agha, Xavier Poire, Martin Carre, Jacques-Olivier Bay, Yves Beguin, Michael Loschi, Anne Huynh, Gaëlle Guillerm, Sylvie François, Jean-Baptiste Mear, Rémy Duléry, Felipe Suarez, Karin Bilger, Jérôme Cornillon, Yves Chalandon, Natacha Maillard, Hélène Labussière-Wallet, Amandine Charbonnier, Pascal Turlure, Ana Berceanu, Sylvain Chantepie, Sébastien Maury, Ali Bazarbachi, Anne-Lise Menard, Stephanie Nguyen-Quoc, Marie-Thérèse Rubio, Maud D’Aveni

**Affiliations:** 1 Hematology department, University hospital of Nancy, Nancy, France; 2 Hematology department, Hôpital Saint-Louis, Paris, France; 3 Biostatistics Consultant, Pérouges, France; 4 Hematology department, Institut Paoli Calmette, Marseille, France; 5 Hematology department, Hôpital UZ Leuven, Louvain, Belgium; 6 Hematology department, University hospital of Nantes, Nantes, France; 7 Hematology department, Institut Gustave Roussy, Paris, France; 8 Hematology department, Hôpital Haut-Levêque, Bordeaux, France; 9 Hematology department, Hôpital Saint Eloi, Montpellier, France; 10 Hematology department, CHU de Lille, Univ Lille, INSERM U1286, Infinite, Lille, France; 11 Section of Hematology, Cliniques Universitaires St-Luc, Brussels, Belgium; 12 Hematology department, Hôpital Michallon, Grenoble, France; 13 Service de Thérapie Cellulaire et d’Hématologie Clinique Adulte, CHU Clermont-Ferrand Hôpital Estaing, Estaing, France; 14 Hematology department, University hospital of Liège, Liège, Belgium; 15 Hematology department Hôpital l’Archet, Nice, France; 16 Hematology department, University hospital of Toulouse, Toulouse, France; 17 Hematology department, University hospital of Brest, Brest, France; 18 Hematology department, University hospital of Angers, Angers, France; 19 Hematology department University hospital of Rennes, Rennes, France; 20 Hematology department, Hôpital Saint-Antoine, Paris, France; 21 Hematology department, Hôpital Necker, Paris, France; 22 Hematology department University hospital of Strasbourg, Strasbourg, France; 23 Hematology department University hospital of Saint-Etienne, Saint-Priest-en-Jarez, France; 24 Hematology division, University hospital of Geneva and Faculty of Medicine, University of Geneva, Geneva, Switzerland; 25 Hematology department University hospital of Poitiers, Poitiers, France; 26 Hematology department, Lyon-Sud Hospital, Lyon, France; 27 Hematology department University hospital of Amiens, Amiens, France; 28 Hematology department, Hôpital Dupuytren, Limoges, France; 29 Hematology department, University hospital of Besançon, Besançon, France; 30 Hematology department, University hospital of Caen, Caen, France; 31 Hematology department, Hôpital Henri Mondor, Créteil, France; 32 Hematology department, American university of Beyrouth, Beyrouth, Lebanon; 33 Hematology department Centre Henri Bequerel, Rouen, France; 34 Hematology department, Hôpital La Pitié Salpêtrière, Paris, France

**Keywords:** Diseases, Myelodysplastic syndrome

## Abstract

Allogeneic hematopoietic stem cell transplantation remains the best curative option for higher-risk myelodysplastic syndrome. The presence of monosomal karyotype and/or complex karyotype abnormalities predicts inferior survival after allo-SCT in MDS patients. Haploidentical allo-SCT has been increasingly used in acute leukemia (AL) and has similar results as using HLA-matched donors, but data on higher-risk MDS is sparse. We compared outcomes in 266 patients with higher-risk MDS after HLA-matched sibling donor (MSD, *n* = 79), HLA-matched unrelated donor (MUD, *n* = 139) and HLA haploidentical donor (HID, *n* = 48) from 2010 to 2019. Median donor age differed between the three groups (*p* < 0.001). The overall survival was significantly different between the three groups with a better OS observed in the MUD group (*p* = 0.014). This observation could be explained by a higher progression-free survival with MUD (*p* = 0.014). The cumulative incidence of grade 2–4 acute GvHD was significantly higher in the HID group (*p* = 0.051). However, in multivariable analysis, patients transplanted using an HID had comparable mortality to patients transplanted using a MUD (subdistribution hazard ratio [sHR]: 0.58 [0.32–1.07]; *p* = 0.080) and a MSD ([sHR]: 0.56 [0.28–1.11]; *p* = 0.094). MUD do not remain a significant positive predictor of survival, suggesting that beyond the donor-recipient HLA matching, the donor age might impact recipient outcome.

## Introduction

Myelodysplastic syndromes (MDSs) constitute a group of heterogeneous clonal hematopoietic stem cell disorders characterized by ineffective hematopoiesis and an increased risk of progression to acute myeloid leukemia (AML) [1]. Allogeneic stem cell transplantation (allo-SCT) remains the only curative treatment by improving survival compared to azacytidine in patients at higher risk according to the International Prognostic Scoring System (IPSS) [2–4]. The revised International Prognostic Scoring System (IPSS-R) published in 2012 [5] improves prognostic ability compared to the IPSS published in 1997 [6] in regard to survival and AML evolution in untreated patients and has demonstrated prognostic significance following allo-SCT [7].

The probability of finding a matched sibling donor (MSD) is estimated to be under the classical 30% because of the age of patients with higher-risk MDS and their relatives [8]. For these patients, a matched unrelated donor (MUD) is considered a valid alternative but can take time to identify. Recently, the Acute Leukemia Working Party (ALWP) of the European Society for Blood and Marrow Transplantation (EBMT) demonstrated similar outcomes after allo-SCT with haploidentical donor (HID) as using an MSD and MUD in high-risk AML [9–11] and acute lymphoid leukemia [12]. However, allo-SCT using an HID in patients with relapsed/refractory AML was associated with inferior outcomes, mainly due to higher non-relapse mortality (NRM) secondary to a high rate of infection [13]. Globally, clinical studies comparing recipient outcomes after allo-SCT with HID *versus* MSD or MUD in myeloid malignancies have suggested similar outcomes, with an overall survival (OS) between 40% and 80% [14, 15]. One prospective study suggested better OS in AML patients with detectable molecular residual disease (MRD) before allo-SCT when using an HID, suggesting a better Graft *versus* Leukemia effect for uncontrolled myeloid malignancy at transplant [16]. Few studies focused on the outcomes after allo-SCT for MDS patients (excluding AML). One study, published in 2016, reported 454 MDS patients who underwent allo-SCT from HIDs (*n* = 226) or MSDs (*n* = 228) in the Chinese Bone Marrow Transplantation Registry [17]. Among the 3/6 HID (*n* = 136), 4–5/6 HID (*n* = 90), and MSD groups, the 4-year adjusted cumulative incidence of NRM was 34%, 29%, and 16%, respectively (overall *p* = 0.004), with a 4-year adjusted probability of OS of 58%, 63%, and 73%, respectively (overall *p* = 0.07), suggesting lower OS in the HID group [14]. Another study published in 2021 [18] enrolled 603 MDS patients transplanted using an HID (*n* = 176) or MUD (*n* = 427) from the Center for International Blood and Marrow Transplant Research database. Multivariate analysis revealed higher relapse (hazard ratio [HR] 1.56; *p* = 0.0055; 2-year relapse rate, 48% vs. 33%) and lower disease-free survival (DFS) rates after allo-SCT with HID (HR 1.29; *p* = 0.042; 2-year DFS, 29% vs. 36%). However, OS rates did not differ between donor type (HR 0.94; *p* = 0.65; 2-year OS, 46% for HID and 44% for MUD) because of the mortality associated with chronic graft *versus* host disease (GvHD) in the MUD group.

Therefore, we decided to conduct a retrospective analysis to investigate the impact of HID *versus* HLA-matched donor (including MSD and MUD) on patient outcomes in allografted MDS. We decided to focus on MDS with higher cytogenetic risks because we know that the malignant clone is rarely controlled before allo-SCT, with the hypothesis that haploidentical SCT better controls the disease, as previously reported in AML [16].

## Material and Methods

### Study design

We conducted a retrospective multicenter study. This study was approved by the scientific committee of the Francophone Society of Bone Marrow Transplantation and Cellular Therapy (SFGM-TC). Informed consent was obtained for each patient before inclusion, and the study was conducted according to the Declaration of Helsinki. Participating centers were asked to verify the recorded data for each patient and to provide additional information.

We included patients transplanted from an HID or HLA-matched donor (10/10) between 2010 and 2019. Inclusion criteria were MDS defined according to the WHO 2008 classification, age ≥18 years, and first allo-SCT. Poor cytogenetics (−7, inv(3)/t(3q)/del(3q), double including −7/del(7q) and 3 abnormalities) and very poor (complex: > 3 abnormalities) according to IPSS-R were included in this study. Exclusion criteria were MDS with very good, good, or intermediate cytogenetics; mismatched unrelated donor (HLA compatibility 9/10) or unrelated cord blood as the stem cell source; standard myeloablative and/or sequential conditioning regimens; and maintenance treatment (e.g., azacytidine) or prophylactic DLI after allo-SCT.

### Patients and transplantation characteristics

IPSS [6] was calculated at diagnosis for each patient. Cytogenetics were stratified based on the MDS Comprehensive Scoring System [19]. Response criteria were defined according to the 2006 International Working Group [20]. Patients received either non-myeloablative, reduced-toxicity conditioning or myeloablative with reduced toxicity regimens.

Patients undergoing myeloablative reduced-toxicity conditioning received FluBu3 or reduced TBF. FluBu3 consisted of fludarabine (150–160 mg/m²) and intravenous (IV) busulfan (3.2 mg/kg daily for 3 days; total dose 9.6 mg/kg). Reduced TBF consisted of thiotepa (5 mg/kg for 1 day), fludarabine (30 mg/m^2^/j; total dose 120 mg/m²), and IV busulfan (3.2 mg/kg for 2 days; total dose 6.4 mg/kg).

Patients undergoing reduced intensity conditioning received FluBu2, CloBu2, or Flu/Mel. FluBu2 consisted of fludarabine (150–160 mg/m²) and IV busulfan (3.2 mg/kg daily for 2 days). CloBu2 consisted of clofarabine (150 mg/m²) with busulfan (3.2 mg/kg for 2 days). Two Gray Total Body Irradiation (TBI) might be added in some patients. Flu/Mel consisted of fludarabine (150–160 mg/m²) and melphalan (100 mg/m²).

Patients undergoing a non-myeloablative regimen received fludarabine (150 mg/m²) or clofarabine (150 mg/m²) with 2 Gy TBI. Cyclophosphamide (29 mg/kg) might be added in some patients.

### Statistical analysis

Descriptive analyses of patient characteristics, treatments, and endpoints were performed for the total population, as well as between the three subgroups based on donor (MSD, MUD, HID). Quantitative variables were summarized as mean and standard deviation if the normality of the distribution was verified by the Lilliefors test, otherwise as median, range, and 1^st^ and 3^rd^ quartiles. Comparisons between groups were achieved using ANOVA or the non-parametric Kruskal-Wallis test according to the distributions. Qualitative variables were summarized as counts and percentages (calculated based on the number of available data) and comparisons between groups achieved using Pearson’s chi-squared (with Monte-Carlo simulations if at least one count was < 5) and Fisher exact tests for endpoints.

The primary outcome of this study was OS, and secondary endpoints included PFS, relapse incidence (RI), NRM, incidence and severity of acute and chronic GvHD, and GvHD relapse-free survival (GRFS). OS and PFS were reported for 2 years. Acute [21] and chronic GvHD [22] were diagnosed and graded using established criteria. OS was defined as the time from stem-cell transplantation to death from any cause or end of follow-up. PFS was defined as the time from stem-cell transplantation to relapse, disease progression, death from any cause, or end of follow-up. RI and NRM were analyzed as competing risks and estimated using cumulative incidence functions (CIFs). The cumulative incidence of grade 2–4 acute GvHD at 100 days and chronic GvHD at 2 years were estimated considering death as a competing event. Univariate analyses of all variables of interest and multivariate analyses studying the impact of HLA matching (including adjustment variables) were performed using the Cox proportional hazard regression for OS, PFS, and GRFS endpoints and the Fine & Gray regression for RI, NRM, and GVH incidence. GRFS was defined as survival without grade III-IV acute GvHD, without chronic GvHD requiring systemic immunosuppressive treatment for severe chronic GvHD, and without relapse [23].

The level of significance was set at 5%. Consequently, estimations of (subdistribution) hazard ratios and probabilities were presented with their 95% bilateral confidence intervals. Statistical analyses and graphics were computed in R v4.1.2 with the help of the ‘survival’, ‘cmprsk’, and ‘ggplot2’ packages.

## Results

### Patient, disease, and transplant characteristics

Patient and transplant characteristics are summarized in Table 1. We included 266 higher-risk MDS patients: 218 patients received allo-SCT from an HLA-matched donor (79 MSD and 139 MUD) and 48 from an HID. Median recipient age at transplant slightly differed between the three groups, with a higher median age of 64.92 years (range 32.86–73.68 years) in the HID group (*p* = 0.019). Median donor age also differed between the three groups, with a higher median age of 59 years (23.65–78.55 years) in the MSD group (*p* < 0.001). There was a trend for a shorter time between diagnosis and transplantation in the MSD group (*p* = 0.065) but no significant difference between the three groups for the duration of follow-up after transplantation (*p* = 0.102). There was no significant difference in terms of MDS diagnosis according to the WHO 2008 classification (*p* = 0.609), IPSS (*p* = 0.453), and cytogenetic risk groups at diagnosis (*p* = 0.521) and status at transplant (*p* = 0.866). As expected, we observed significant differences between the three groups in terms of conditioning regimen (*p* < 0.001), stem cell source (*p* < 0.001), in vivo T-cell depletion (*p* < 0.001), and GvHD prophylaxis (*p* < 0.001), which are linked to the backbone of the haploidentical allo-SCT platform.

**Table 1:** Patient and transplant characteristics (N = 266).

Number of patients	MSD	MUD	HID	*p*-value
	79 (29.70%)	139 (52.26%)	48 (18.05%)	
Recipient age at diagnosis (years), median (range)	60.08 (28.75–69.63)	62.14 (28.1–73.93)	64.01 (32.33–72.17)	**0.036**
Recipient age at transplant (years), median (range)	60.62(29.24–73.89)	62.68(28.35–74.45)	64.92 (32.86–73.68)	**0.019**
Donor age (years), median (range)	59 (23.65–78.55)	29.74 (19.27–55.11)	39.98 (18.29–69.64)	**<0.001**
Time from diagnosis to transplant (months), median (range)	6 (0; 53)	7 (1; 64)	8 (3–87)	0.065
Follow-up (months), median (range)	12.09 (0–114.89)	13.77 (0–120.1)	6.78 (0.46–72.57)	0.102
Percentage of marrow blasts at diagnostic, median (range)	7 (0–19)	8 (0–19)	6.5 (0–16)	0.470
Percentage of marrow blast at transplant, median (range)	3 (0–19)	2 (0–19)	2 (0–12)	0.438
MDS according to WHO classification at diagnostic				0.609
RAEB-1	19/79 (24.05%)	34/139 (24.46%)	16/48 (33.33%)	
RAEB-2	32/79 (40.51%)	53/139 (38.13%)	13/48 (27.08%)	
RA, RARS, RCMD	18/79 (22.78%)	40/139 (28.78%)	13/48 (27.08%)	
Unclassifiable, other	10/79 (12.66%)	12/139 (8.63%)	6/48 (12.50%)	
IPSS at diagnosis, *n* (%)				0.454
High (> 2.5)	31/76 (40.79%)	51/133 (38.35%)	18/47(36.17%)	
Intermediate-2 (1.5–2)	37/76 (48.68%)	59/133 (44.36%)	19/47 (40.43%)	
Intermediate-1 (0.5–1)	8/76 (10.53%)	23/133 (17.29%)	11/47 (23.40%)	
Cytogenetic prognosis according to R-IPSS				0.521
Very High	29/79 (36.71%)	48/139 (34.53%)	21/48 (43.75%)	
High	50/79 (63.29%)	91/139 (65.47%)	27/48 (56.25%)	
Number of lines before transplantation				0.594
0	26/79 (32.91%)	37/139 (26.62%)	13/48 (27.08%)	
At least 1	53/79 (67.09%)	102/139 (73.38%)	35/48 (72.92%)	
Status at transplant (according to IWG 2006)				0.866
Complete remission (CR)	36/79 (45.57%)	59/139 (42.45%)	19/48 (39.58%)	
Response without CR	15/79 (18.99%)	27/139 (19.42%)	10/48 (20.83%)	
Stable disease	19/79 (24.05%)	36/139 (25.90%)	14/48 (29.17%)	
Progression	9/79 (11.39%)	12/139 (8.63%)	4/48 (8.33%)	
Not evaluable	0/79 (0.00%)	5/139 (3.60%)	1/48 (2.08%)	
Comorbidity index				
Number of patients with available data	65/79 (82.27%)	111/139 (79.85%)	42/48 (87.5%)	
Median (min-max)	2 (0–6)	2 (0–9)	1 (0–6)	0.341
Conditioning regimens				**<0.001**
RIC	60/79 (75.95%)	109/138 (78.99%)	3/48 (8.33%)	
NMA	3/79 (3.80%)	0/138 (0.00%)	23/48 (47.92%)	
RTC	16/79 (20.25%)	29/138 (21.01%)	21/48 (43.75%)	
Stem cell source				**<0.001**
Bone marrow	4/79 (5.06%)	9/139 (6.47%)	15/48 (31.25%)	
Peripheral blood stem cell	75/79 (94.94%)	130/139 (93.53%)	33/48 (68.75%)	
Donor/recipient sex match				**<0.001**
Female donor/male recipient	28/79 (35.44%)	14/139 (10.07%)	8/48 (16.67%)	
Others	51/79 (64.56%)	125/139 (89.93%)	40/48 (83.33%)	
CMV risk				**0.037**
High-risk (seronegative donor, seropositive recipient)	20/76 (26.32%)	33/135 (24.44%)	13/48 (27.08%)	
Intermediate-risk (seropositive donor)	43/76 (56.58%)	54/135 (40.00%)	25/48 (52.08%)	
Low-risk (seronegative donor, seronegative recipient)	13/76 (17.11%)	48/135 (35.56%)	10/48 (20.83%)	
In vivo T-cell depletion, n/N (%)	67/79 (84.81%)	130/139 (93.52%)	48/48 (100%)	**<0.001**
ATG	67/79 (84.81%)	130/139 (93.53%)	8/48 (16.67%)	
Post-transplant cyclophosphamide			45/48 (93.75%)	
GvHD prophylaxis, n/N (%)				**<0.001**
Ciclosporin/Tacrolimus alone	27/78 (34.92%)	21/138 (15.22%)	4/48 (8.33%)	
Ciclosporin/Tacrolimus + MMF	36/78 (46.15%)	76/138 (55.07%)	43/48 (89.58%)	
Ciclosporin/Tacrolimus + MTX	13/78 (16.67%)	38/138 (27.54%)	1/48 (2.08%)	
Other	2/78 (2.56%)	3/138 (2.17%)	0/48 (0.00%)	

*ATG* Anti-thymocyte globulin, *CMV* Cytomegalovirus, *CR* Complete remission, GvHD Graft *versus* host disease, *HID* Haploidentical donor, *IPSS* International prognostic scoring system, *IPSS-R* Revised international prognostic scoring system, *MMF* Mycophenolate mofetil, *MTX* Methotrexate, *MSD* Matched sibling donor, *MUD* Matched unrelated donor, *NMA* Non-myeloablative, *RA* Refractory Anemia, *RAEB-1* Refractory Anemia with Excess Blasts 1, *RAEB-2* Refractory Anemia with Excess Blasts 2, *RARS* Refractory Anemia with Ring Sideroblasts, *RCMD* Refractory Cytopenia with Multilineage Dysplasia, *RIC* Reduced-intensity conditioning, *RTC* Reduced-toxicity conditioning.

Statistically significant *p* < 0.05 values are in bold.

### Overall outcomes

Overall outcomes are summarized in Table 2. Engraftment was comparable between the three groups (*p* = 0.119). Three cases of secondary graft rejection occurred in the haploidentical group (6.25%) and four cases in the MUD group (2.88%). In all groups, the main cause of death was relapse or progression of the original disease (MSD: 67.27%; MUD: 48.15%; HID: 48.65%; *p* = 0.338).

**Table 2:** Patient outcomes (*N* = 266).

	MSD		MUD		HID		*p*-value*
	*N* = 79		*N* = 139		*N* = 48		
	n/N	%	n/N	%	n/N	%	
**Engrafment**							0.119
Full donor	65/79	82.27	117/139	84.17	34/48	70.83	
Mixed	8/79	10.13	12/139	8.63	5/48	10.42	
Graft rejection	0/79	0.00	3/139	2.15	3/48	6.25	
Missing	6/79	7.60	7/139	5.05	6/48	12.50	
**Acute GvHD**							
Grade 0-1	59/79	74.68	87/139	62.59	27/48	56.25	0.073
Grade 2-4	17/79	21.52	49/139	35.25	19/48	39.58	**0.051**
Grade 3-4	8/79	10.13	21/139	15.11	8/48	16.67	0.493
**Chronic GvHD**							0.342
No	53/78	67.95	90/137	65.69	37/48	77.08	
Yes	25/78	32.05	47/137	34.31	11/48	22.92	
**Score of chronic GvHD**							0.288
Limited	14/25	56.00	25/47	53.19	3/11	27.27	
Extensive	11/25	44.00	22/47	46.81	8/11	72.73	
**Survival status**							0.028
Dead	55/79	69.62	80/139	57.55	37/48	77.08	
Alive	24/79	30.38	59/139	42.45	11/48	22.92	
**Main causes of death**							0.338
Relapse or progression	37/55	67.27	31/80	48.15	18/37	48.65	
GvHD	6/55	10.91	19/80	23.75	10/37	27.03	
Infection	4/55	7.27	10/80	12.50	5/37	13.51	
Hemorrhage	0/55	0.00	3/80	3.75	0/37	0.00	
Multiple organ failure	2/55	3.64	1/80	1.25	1/37	2.86	
VOD	1/55	1.82	0/80	0.00	0/37	0.00	
Other causes related to SCT	3/55	5.45	7/80	8.75	3/37	8.11	
Secondary malignancy	2/55	3.64	2/80	2.50	0/37	0.00	

*GvHD* Graft *versus* host disease, *HID* Haploidentical donor, *MSD* Matched sibling donor, *MUD* Matched unrelated donor, *SCT* Stem cell transplantation, *VOD* Veno-occlusive disease.

Statistically significant *p* < 0.05 values are in bold.

The cumulative incidence of grade II-IV acute GvHD on day 100 was 18.99% [95% CI: 10.28–27.70%] in the MSD group, 34.31% [95% CI: 26.32–42.29%] in the MUD group, and 37.50% [95% CI: 23.60–51.40%] in the HID group (*p* = 0.036, Table 3).

**Table 3:** Survival (OS, PFS, and GRFS) and cumulative incidences of NRM, RI, and GvHD according to HLA matching at one, two and three years after allo-SCT.

	MSD		MUD		HID	
At one year	%	95%CI	%	95%CI	%	95%CI
OS	54.78	[44.56–67.34]	58.88	[51.08–67.87]	35.42	[24.71–51.89]
PFS	39.97	[30.30–52.74]	50.20	[42.38–59.46]	31.25	[20.54–47.54]
GRFS	31.91	[22.90–44.48]	35.47	[28.18–44.64]	27.08	[17.03–43.08]
aGvHD grade 2–4	18.99	[10.28–27.70]	34.31	[26.32–42.29]	37.50	[23.60–51.40]
cGvHD	29.44	[18.97–39.91]	31.19	[23.22–39.16]	22.92	[10.81–35.03]
NRM	19.58	[10.59–28.56]	18.57	[11.96–25.19]	33.33	[19.76–46.91]
RI	40.45	[29.29–51.61]	31.23	[23.33–39.12]	35.42	[21.65–49.18]
**At Two years**						
OS	34.99	[25.32–48.34]	47.18	[39.24–56.71]	23.77	[14.04–40.22]
PFS	28.28	[19.34–41.36]	43.10	[35.29–52.64]	20.00	[11.20–35.71]
GRFS	20.37	[12.69–32.70]	29.48	[22.54–38.55]	15.62	[7.91–30.88]
cGvHD	31.06	[20.34–41.78]	37.77	[29.33–46.20]	25.00	[12.49–37.51]
NRM	21.35	[11.88–30.81]	21.98	[14.83–29.12]	33.33	[19.76–46.91]
RI	50.38	[38.50–62.25]	34.92	[26.65–43.19]	46.67	[31.96–61.37]
**At Three years**						
OS	26.66	[17.83–39.85]	42.25	[34.32–52.02]	19.80	[10.48–37.42]
PFS	21.21	[13.21–34.07]	40.12	[32.33–49.78]	20.00	[11.20–35.71]
GRFS	13.58	[7.31–25.24]	27.51	[20.69–36.58]	15.62	[7.91–30.88]
cGvHD	32.67	[22.72–43.63]	37.77	[29.33–46.20]	25.00	[12.49–37.51]
NRM	21.35	[11.88–30.81]	23.99	[16.50–31.47]	33.33	[19.76–46.91]
RI	57.45	[45.43–69.46]	35.90	[27.53–44.27]	46.67	[21.65–49.18]

*GRFS* GvHD and relapse free survival, *GvHD* Graft versus host disease (*aGvHD* Acute Graft versus host disease, *cGvHD* Chronic Graft versus host disease), *HID* Haploidentical donor, *MSD* Matched sibling donor, *MUD* Matched unrelated donor, *NRM* Non relapse mortality, *OS* Overall survival, *PFS* Progression free survival, *RI* Relapse incidence.

Cumulative incidence of chronic GvHD 12 months after allo-SCT was 29.44% [95% CI: 18.97–39.91%] in the MSD group, 31.19% [95% CI: 23.22–39.16%] in the MUD group, and 22.92% [95% CI: 10.81–35.03%] in the HID group (*p* = 0.486, Table 3).

**Fig. 1:**
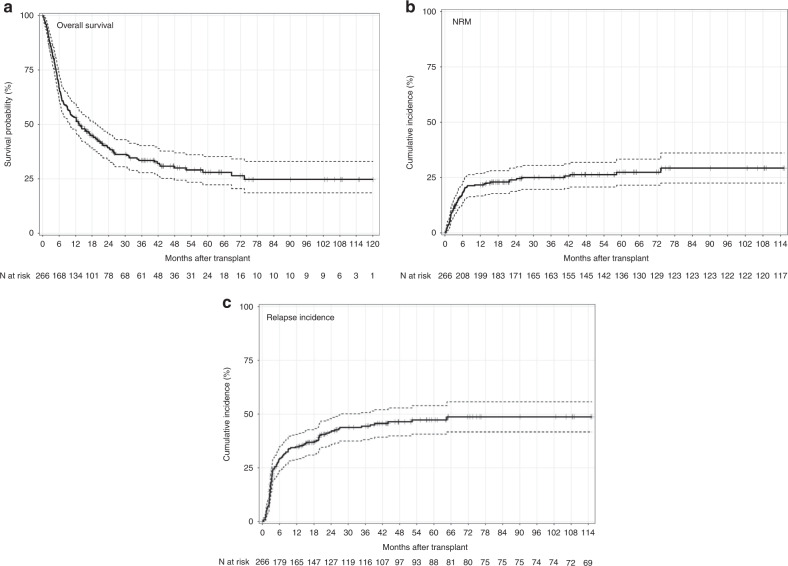
Transplant outcomes (*N* = 266). **a** Overall survival. **b** Non relapse mortality (NRM). **c** Cumulative incidence of relapse.

**Fig. 2:**
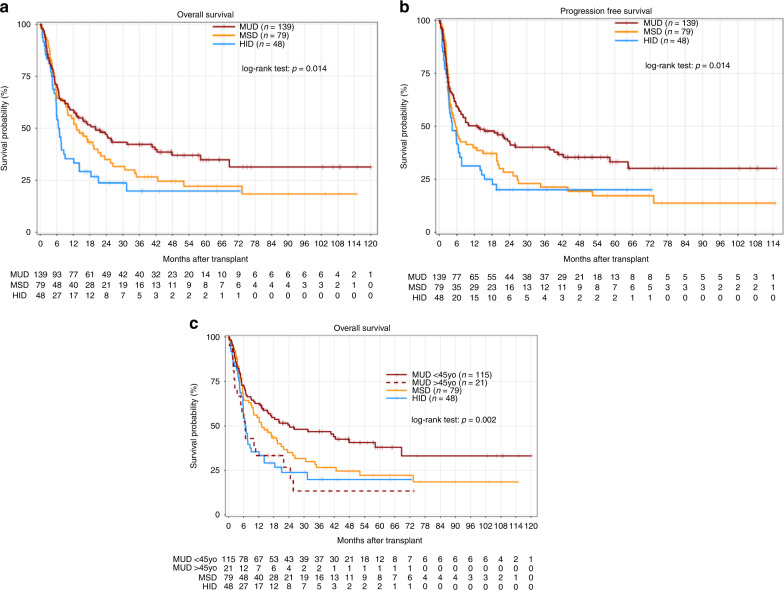
Transplant outcomes according to donor. **a** Overall survival. **b** Progression free survival. **c** Better overall survival for recipients transplanted with younger matched unrelated donor.

In the entire cohort, very early after allo-SCT, the 1-year estimated outcomes were poor, with an OS of 39.27% [95% CI: 33.56- 45.95%] and a 1-year NRM of 23.94% [95% CI:18.66–29.23%]. The poor outcomes are in accordance with a very high 1-year relapse incidence of 41.66% [95% CI: 35.48–47.84%] in agreement with the initial disease characteristics (Table 3 and Fig. 1a-c). In this study, we observed that only the OS (*p* = 0.014) and PFS curves (*p* = 0.014) were significantly different between the three groups (Fig. 2a, b). Of note, the OS was significantly higher for patients transplanted with a young MUD ( < 45 years old); *p* = 0.002 (Fig. 2c). Notably, NRM, CIR, and GRFS were comparable between the three groups (data not shown).

### Univariate and multivariate analyses

Factors associated with event occurrence are summarized in Table 4.

**Table 4:** Univariate and multivariate analyses.

*	Mortality (inverse OS)				Treatment failure (inverse of PFS)				Non relapse mortality				Relapse incidence			
	Univariate analysis		Multivariate analysis		Univariate analysis		Multivariate analysis		Univariate analysis		Multivariate analysis		Univariate analysis		Multivariate analysis	
	HR [95% CI]	*p-*value	HR [95% CI]	*p*-value	HR [95% CI]	*p*-value	HR [95% CI]	*p*-value	HR [95% CI]	*p*-value	HR [95% CI]	*p*-value	HR [95% CI]	*p*-value	HR [95% CI]	*p*-value
HLA matching																
HID	1.00		1.00		1.00		1.00		1.00		1.00		1.00		1.00	
MUD	**0.56 [0.38–0.84]**	**0.004**	0.58 [0.32–1.07]	0.080	**0.59 [0.40–0.86]**	**0.007**	0.64 [0.35–1.17]	0.145	0.68 [0.38–1.24]	0.210	0.82 [0.30–2.23]	0.690	0.75 [0.45–1.24]	0.260	0.63 [0.30–1.30]	0.210
MSD	0.73 [0.48–1.11]	0.137	0.56 [0.28–1.11]	0.094	0.82 [0.55–1.23]	0.341	0.69 [0.35–1.36]	0.282	0.59 [0.30–1.19]	0.140	0.58 [0.17–1.95]	0.370	1.21 [0.73–2.00]	0.460	0.83 [0.38–1.82]	0.650
Recipient age	1.01 [0.99–1.03]	0.457	1.01 [0.98–1.03]	0.616	1.01 [0.99–1.02]	0.545	1.01 [0.99–1.03]	0.603	1.01 [0.98–1.04]	0.600	1.00 [0.97–1.04]	0.840	1.00 [0.98–1.02]	0.980	1.00 [0.97–1.03]	1.000
Donor age	**1.01 [1.00–1.02]**	**0.028**	1.01 [1.00–1.03]	0.117	**1.01 [1.00**–**1.02]**	**0.010**	1.01 [0.99–1.03]	0.214	1.00 [0.99–1.02]	0.880	1.01 [0.98–1.04]	0.450	**1.01 [1.00**–**1.03]**	**0.015**	1.01 [0.99–1.03]	0.560
Conditioning regimen																
RIC	1.00		1.00		1.00		1.00		1.00		1.00		1.00		1.00	
NMA	1.49 [0.91–2.46]	0.117	0.92 [0.45–1.86]	0.810	1.45 [0.89–2.35]	0.137	1.01 [0.50–2.01]	0.986	2.10 [1.03–4.30]	0.051	2.32 [0.77–7.00]	0.140	0.76 [0.36–1.59]	0.470	0.44 [0.18–1.09]	0.076
RTC	1.04 [0.73–1.47]	0.840	0.98 [0.64–1.48]	0.909	0.98 [0.70–1.38]	0.913	0.95 [0.63–1.43]	0.813	1.44 [0.85–2.46]	0.180	1.42 [0.77–2.62]	0.270	0.79 [0.51–1.21]	0.280	0.68 [0.36–1.19]	0.180
GvHD prophylaxis																
CI alone	1.00		1.00		1.00		1.00		1.00		1.00		1.00		1.00	
CI + MMF	1.32 [0.88–1.96]	0.181	1.15 [0.74–1.77]	0.544	1.26 [0.86–1.85]	0.232	1.16 [0.76–1.78]	0.486	1.08 [0.57–2.06]	0.820	0.98 [0.45–2.13]	0.960	1.18 [0.76–1.82]	0.460	1.13 [0.72–1.77]	0.580
CI + MTX	1.12 [0.68–1.83]	0.665	1.22 [0.71–2.08]	0.470	1.11 [0.69–1.77]	0.678	1.27 [0.76– 2.12]	0.359	1.30 [0.61–2.77]	0.490	1.40 [0.59–3.34]	0.450	0.87 [0.49–1.56]	0.650	0.90 [0.48–1.69]	0.740
Sex match																
Other combinations	1.00		1.00		1.00		1.00		1.00		1.00		1.00		1.00	
Female to male	0.99 [0.67–1.45]	0.954	0.83 [0.54–1.26]	0.379	0.90 [0.61–1.32]	0.588	0.73 [0.48–1.12]	0.147	1.18 [0.66–2.10]	0.580	1.33 [0.70–2.52]	0.390	0.77 [0.47–1.27]	0.300	0.56 [0.32–0.99]	**0.047**
Stem cell source																
BM	1.00		1.00		1.00		1.00		1.00		1.00		1.00		1.00	
PBSC	1.04 [0.63–1.72]	0.867	1.25 [0.70–2.22]	0.448	1.08 [0.66–1.78]	0.760	1.17 [0.66–2.06]	0.588	1.91 [0.68–5.34]	0.220	2.94 [0.90–9.61]	0.074	0.83 [0.46–1.50]	0.530	0.61 [0.32–1.17]	0.140
CMV risk																
Low	1.00		1.00		1.00		1.00		1.00		1.00		1.00		1.00	
Intermediate	1.32 [0.91–1.91]	0.144	1.09 [0.73–1.62]	0.674	**1.48 [1.02–2.14]**	**0.037**	1.23 [0.83–1.82]	0.310	1.03 [0.59–1.79]	0.930	1.03 [0.56–1.89]	0.920	**1.66 [1.02–2.70]**	**0.043**	1.37 [0.80–2.33]	0.250
High	0.85 [0.55–1.32]	0.474	0.84 [0.53–1.33]	0.446	1.04 [0.68–1.59]	0.865	1.03 [0.66–1.61]	0.902	0.60 [0.29–1.23]	0.160	0.58 [0.27–1.26]	0.170	1.55 [0.90–2.67]	0.120	1.48 [0.84–2.60]	0.180

*BM* Bone marrow, *CMV* Cytomegalovirus, *CI* Calcineurin inhibitor, *GvHD* Graft versus host disease, *HID* Haploidentical donor, *MMF* Mycophenolate mofetil, *MTX* Methotrexate, *MSD* Matched sibling donor, *MUD* Matched unrelated donor, *NMA* Non-myeloablative, *PBSC* Peripherical blood stem cell, *RIC* Reduced intensity conditioning, *RTC* Reduced-toxicity conditioning.

Statistically significant *p* < 0.05 values are in bold.

In univariate analysis, factors predicting OS were the type of donor, with an HR of 0.56 for MUD (*p* = 0.004), and donor age considered as a continuous variable (HR:1.01, *p* = 0.028). Similarly, factors predicting PFS were the type of donor, with an HR of 0.59 for MUD (*p* = 0.007), and donor age considered as a continuous variable (HR: 1.01, *p* = 0.010).

In multivariable analysis, none of the factors (HLA matching, recipient age at transplant, conditioning regimen, GvHD prophylaxis, stem cell source, and CMV risk) were associated with GRFS or chronic GvHD, which is why GRFS and chronic GvHD are not represented. Patients transplanted using an HID had comparable mortality to patients transplanted using a MUD ([sHR]: 0.58 [0.32–1.07]; *p* = 0.080) or a MSD ([sHR]: 0.56 [0.28–1.11]; *p* = 0.094). We observe a similar treatment failure (i.e., inverse of PFS) in the MUD group ([sHR]: 0.64 [0.35–1.17]; p = 0.145) and in the MSD group ([sHR]: 0.69 [0.35–1.36]; *p* = 0.282) compared to the HID group. Also, we observe a similar NRM in the MUD group ([sHR]:0.82 [0.30–2.23]; *p* = 0.690) and in the MSD group ([sHR]: 0.58 [0.17–1.95]; *p* = 0.370) compared to the HID group.

MUD do not remain a significant positive predictor of survival in multivariate analysis, suggesting that the significantly different donor age between the three groups might be a confounding factor at transplant.

## Discussion

Allo-SCT remains the only curative treatment for higher-risk MDS. Unfortunately, available matched sibling donors are rare due to the older age of patients and their relatives. Though retrospective and prospective studies of AML have demonstrated that survival after allo-SCT performed using an HID is globally comparable to survival after allo-SCT using a MUD or MSD [11, 13], data are lacking for MDS patients excluding AML. A recent meta-analysis suggested similar outcomes between allo-SCT performed using a MSD, MUD, or HID, but the risk of developing grade 2–4 GvHD was significantly higher with an HID than a MSD, with a pooled odd ratio of 2.32 [24]. The main conclusion was that the donor type seems to not be a significant determinant of OS, PFS, NRM, or relapse incidence. Notably, the meta-analysis mainly included studies considering AML patients, with a very small proportion of patients with all-risk MDS. Therefore, we decided to focus on MDS (excluding AML) patient outcomes after allo-SCT, especially the poor and very poor risk cytogenetic categories. According to the IPSS-R [5], the presence of complex karyotype abnormalities, monosomal karyotype, or both predicts inferior survival after allo-SCT in MDS patients [25, 26]. TP53 deletion or mutation (alone or in association) is associated with poorer outcomes, with a high risk of mortality and a higher risk of relapse [27]. Regarding the results of one prospective study suggesting better OS of AML patients with a detectable MRD before allo-SCT when using an HID [16], we hypothesized that HID could provide, early after transplantation, a better GVL control for MDS with very high risk of relapse.

Our study demonstrates, in this specific “high-risk cohort”, a low estimated 1-year OS of approximately 40% because of a high relapse incidence (~40%), suggesting that improvements in strategies for relapse control after transplantation are still needed. Moreover, our univariate analysis demonstrated a lower risk of mortality, with a lower treatment failure after allo-SCT using a MUD compared to an HID. One explanation may be that early immune recovery provided better outcomes after SCT [28] and α/β T-cells, NK cells and monocyte reconstitution is delayed after haplo-SCT with post-transplant cyclophosphamide (PTCy) [29].

Our results are in accordance with Grunwald et al. [18]. They demonstrated a higher relapse rate (HR 1.56; *p* = 0.0055; 2-year relapse rate, 48% vs. 33%) and lower PFS rate after allo-SCT using an HID compared to a MUD (HR 1.29; *p* = 0.042; 2-year PFS, 29% vs. 36%). However, in their study, the OS did not differ between the two donor types (HR 0.94; *p* = 0.65; 2-year OS, 46% for HID and 44% for MUD) because of higher mortality associated with chronic GvHD in the MUD group. In our study, we did not observe any difference in the univariate and multivariate analyses of chronic GvHD incidence according to donor type. In the study by Grunwald et al. [18], recipients of HLA-haploidentical donor transplantations received uniform GvHD prophylaxis consisting of PTCy with calcineurin inhibitor and mycophenolate, and recipients of MUD transplantations received GvHD prophylaxis that included calcineurin inhibitor with methotrexate or mycophenolate, but neither treatment group received antithymocyte globulin (ATG) or alemtuzumab. In our study, 93.53% of recipients of MUD transplantations received GvHD prophylaxis that included calcineurin inhibitor with methotrexate or mycophenolate and ATG. The use of ATG has been related to a lower risk of chronic GvHD in prospective randomized trials and, therefore, may explain why recipients transplanted using an MUD in our study did not have excess chronic GvHD. Moreover, recipients in Grunwald et al. were clearly older than the recipients in our study, as they focused on recipients transplanted between the age of 50 and 79 years, which could impact the incidence of chronic GvHD.

In contrast, our results are different from those published by the Chinese Bone Marrow Transplantation Registry [17]. In this study, myeloablative conditioning was homogeneously administered to patients with high doses of cytarabine, busulfan, cyclophosphamide, and semustine. Moreover, GvHD prophylaxis differed with rabbit ATG for the HID group and, notably, no PTCy. Myeloablative regimens were excluded in our study to account for potential differences in NRM and to avoid higher heterogeneity in the conditioning regimens. Moreover, to date, intensifying conditioning regimen have not demonstrated better outcomes for MDS patients [30, 31].

One major limitation of our study is the heterogeneity of the conditioning regimens depending on each center’s policy, even if we tried to gather them into three main groups. The second main limitation is that practitioners may extend GvHD prophylaxis in patients with HIDs, but data on immunosuppressant tapering were not provided by the Promise database. It has been demonstrated that early tapering of immunosuppressive agents can improve the survival of patients with advanced acute myeloid leukemia, and this strategy might be of benefit for high-risk MDS. The third limitation is the imbalanced characteristics between the groups at transplant in terms of graft type, conditioning regimen, and GvHD prophylaxis. However, the three groups were comparable in regard to the MDS characteristics. Because the main differences between the three groups were intrinsically linked to the backbone of the haploidentical allo-SCT platform, we did not choose to perform a propensity score analysis and focused, instead, on the multivariate analysis. Despite the limitations of this study, our results are in agreement with a recent report in AML patients [32]. They compared the recipient outcomes after allo-SCT performed using HIDs *versus* MUDs with similar conditioning and GvHD prophylaxis platforms (RIC and PTCy/calcineurin inhibitor/mycophenolate mofetil, respectively). In this cohort, they also observed lower PFS and OS after allo-SCT in the HID group *vs*. MUD group.

Finally, in agreement with Raj et al. studies in MDS, that include donor kinship are needed [33].

In conclusion, our study demonstrated similar outcomes after allo-SCT with haploidentical donor (HID) as using an MSD and MUD in high-risk MDS. Also, we suggest that the effect of increasing donor age in the MUD group is detrimental to overall survival. Large prospective trials are required to confirm these results.

## Data Availability

All data supporting this article are provided in the manuscript (Tables 1, 2, 3 and 4 and Figs. 1 and 2).
